# Flavonoids in Ecuadorian* Oreocallis grandiflora* (Lam.) R. Br.: Perspectives of Use of This Species as a Food Supplement

**DOI:** 10.1155/2018/1353129

**Published:** 2018-12-11

**Authors:** Diego Vinueza, Karina Yanza, Massimo Tacchini, Alessandro Grandini, Gianni Sacchetti, Matteo Andrea Chiurato, Alessandra Guerrini

**Affiliations:** ^1^Natural Products Laboratory, Sciences Faculty, Polytechnic School of Chimborazo, Panamericana Sur km 1 ½, CP 060155, Riobamba, Ecuador; ^2^Department of Life Sciences and Biotechnology (SVeB,) University of Ferrara, Via Conca, Piazzale Chiappini 3, 44123 Ferrara (fraz. Malborghetto di Boara), Italy

## Abstract

*Oreocallis grandiflora *(Lam.) R. Br. is an Ecuadorian species belonging to the Proteaceae family, commonly known as* cucharillo* (Loja and Zamora provinces),* cucharilla* (Sierra region),* gañal* (Bolívar province), and* algil *(Chimborazo province). Its leaves and flowers, collected during blooming, are traditionally used for oral administration to treat liver diseases, vaginal bleeding, and ovary/uterus inflammation and as digestive, diuretic, and hypoglycemic remedy. Related literature does not report any scientific evidences regarding the chemical composition of the used parts of this species (leaves and flowers), while few indications are reported about the healthy properties of their preparations. Based on these premises, the present research was performed with the objectives to fill the gaps of the chemical and biological knowledge about this species, enriching the knowledge related to the plant biodiversity of Amazonian Ecuador and to the ethnobotanical tradition of Andean communities. Chemical and biological investigation (*in vitro* antioxidant and anti-inflammatory activity) of flower and leaf hydroalcoholic extracts shed a light on the functional metabolites putatively involved in healthy properties of the* O. grandiflora *traditional preparations. The chemical fingerprinting achieved by HPTLC and ^1^HNMR analyses showed the presence of flavonoids, subsequently quantitatively estimated by AlCl_3_ complexation assay and HPLC-DAD. Silica gel chromatography allowed the isolation of the main compounds of the flower extract: quercetin 3-O-*β*-glucuronide and myricetin 3-O-*β*-glucuronide. RP-HPLC-DAD-MS analyses showed the presence of quercetin 3-O-rutinoside and isorhamnetin 3-O-rutinoside, in addition to the above-mentioned molecules, in the leaf extract. Regarding the antioxidant (DPPH test, a radical scavenging assay) and anti-inflammatory (WST-1 assay, an oxidative burst test) activities, leaf extract showed the most promising results when compared to the positive controls. The same extract, however, exhibited a higher cytotoxicity compared to the flower extract, indicating the latter preparation as the most interesting anti-inflammatory crude drug.

## 1. Introduction

The Proteaceae family is one of the most prominent of the southern hemisphere and consists of 79 genera and over 1700 species. It has a main diversity center in Australia and secondary centers in South Africa, New Caledonia, Southeast Asia, Madagascar, New Zealand, and South America. This family, apparently of tropical origin, is characterized by a wide spectrum of variation in vegetative morphology (Proteaceae take the name from the Greek god Proteus, who had the ability to change between many forms). However, according to the latest botanical systematic profile of this family based on traditional classification parameters, eight genera of Proteaceae can be found in South America, all within the subfamily Grevilleoideae Engl.; six of them are endemic to South America and the other two occur also in Australia and Tasmania [1].

*Oreocallis grandiflora *(Lam.) R. Br. (OG) is an Ecuadorian species belonging to the Proteaceae family, commonly known as* cucharillo* (Loja and Zamora provinces),* cucharilla* (Sierra region),* gañal* (Bolívar province), and* algil* (Chimborazo province). Its leaves and flowers, collected during blooming, are traditionally used for oral administration to treat liver disease, vaginal bleeding, and ovary/uterus inflammation and as digestive, diuretic, and hypoglycemic remedy [2–7]. These traditional uses drove our research, mainly focused on valorizing Andean plant biodiversity and plant health uses heritage, to study the chemical composition and the biological activities of flower and leaf hydroalcoholic extracts of OG.

To the best of our knowledge, the related literature does not report any scientific papers regarding the chemical composition of the used parts of this species, while few indications refer to the healthy properties of their preparations [6]. Some other Proteaceae species have instead been studied for the chemical composition of their used parts. For example,* Grevillea robusta* showed interesting amounts of flavonoids [8], likewise a study on* Helicia excelsa *Roxb. indicated the presence of flavonoids, saponins, terpenes, and tannins [9], and* Hakea salicifolia* and* Hakea sericea *were reported to contain phenolic and flavonoids compounds and smaller amounts of terpenes [10].

Based on these premises, the present research was conducted with the objectives to fill the gaps of the chemical and biological knowledge about this species, which is important for both Ecuadorian plant biodiversity and traditional ethnomedical uses of Andean plants, by investigating the chemical composition and bioactivity (*in vitro* antioxidant, anti-inflammatory, and cytotoxic properties) of flower and leaf hydroalcoholic extracts for the presence of functional metabolites putatively involved in healthy properties of the OG traditional preparations. The importance of profiling Andean plants for their chemical and biological properties would valorize the knowledge about the botanical heritage of one of the most important biodiversity hot-spot in the world and contemporarily to shed a light on the opportunity to enrich the food supplements global market of new plant source of bioactive constituents: this paper goes towards this direction.

## 2. Materials and Methods

### 2.1. Materials

Ficoll Paque, modified Hank's solution, Zimosan A, Triton X-100,trolox, quercetin, gallic acid, DPPH, natural product reagent A, polyethylene glycol, ethyl acetate, acetic acid, formic acid, acetonitrile, methanol, deuterated methanol, and ethanol were purchased from Sigma-Aldrich, SL (USA), water soluble tetrazolium salt (WST-1) from Roche (USA), aspirin from J.T. Baker (USA), dimethyl sulfoxide (DMSO) and ammonium chloride from Merck (Germany), and quercetin 3-O-*β*-rutinoside (rutin), quercetin 3-O-*β*-glucuronide (miquelianin), and isorhamnetin 3-O-*β*-rutinoside (narcissin) from Extrasynthese (France); deionized water was used in all experimental procedures. All other reagents and solvents were of analytical or high-performance liquid chromatography grade as appropriate.

### 2.2. Collection of Plant Material

*O. grandiflora* (Lam.) R. Br., Proteaceae, plants, herein after referred to as OG, were collected in Ecuador, Loja province, Olmedo town, sector S 03° 56′ 0.172′′ W 079° 38′ 25.814′′, at 1314 meters above sea level, during full blossom (balsamic period). The plant material was taxonomically identified by the botanist Jorge Caranqui at Escuela Superior Politécnica de Chimborazo (ESPOCH) and a specimen was deposited at ESPOCH's Herbarium (code: ESPOCH 4323).* O. grandiflora* leaves and flowers were collected at balsamic period, dried at 50°C in a forced convection oven for 8 h, and then separately ground in a knife mill until particle size of 2-3 mm.

### 2.3. Extraction Procedures

The dried powdered flowers and leaves (100 g each) were extracted by maceration with 1000 mL of 70% v/v ethanol for 72 h at room temperature and kept mixed in an orbital shaker to 100 rpm. Then, the extracts were filtered and the process was repeated on the marc until material was exhausted. The collected filtrates were pooled and evaporated under reduced pressure (50°C, -0.5 bar) to obtain the dry extracts (3.02% and 1.82% for yield of leaves and flowers, respectively). The solids were stored at 4°C and vacuum until use.

### 2.4. HPTLC Screening

HPTLC (high-performance thin layer chromatography) was assessed to determine the main compounds of the two extracts. Two microliters of a hydroalcoholic solution of OG extracts (10 mg/mL) were applied to a HPTLC silica gel 60 F254 plate (Merck, Germany) as 6 mm wide bands with Linomat V (Camag, Switzerland). Then, the plate was eluted with a solvent solution (ethyl acetate/acetic acid/formic acid/water 10/0.5/0.5/2) in a chromatographic chamber. After development, the chromatogram was visualized at 254 nm and then was sprayed with natural product reagent A polyethylene glycol (NP/PEG) reagent and visualized at 366 nm to identify the phenolic fingerprinting of the two extracts. The flavonoids appeared as yellow-orange areas on a violet background [11].

### 2.5. NMR

The ^1^H- and ^13^C-, DEPT-NMR, and 2D-NMR spectra (COSY, HMQC, HMBC) were recorded in CD_3_OD solution in 5 mm tubes, at room temperature, with a Varian Mercury Plus 400, operating at 400 (1H) and 100 MHz (13C), respectively; the ^1^H and ^13^C chemical shifts referenced to the residual solvent signal (CD_3_OD: *δ* (H) 3.34 ppm and, *δ*(C) 49.0 ppm, respectively).

### 2.6. Total Flavonoids Determination

The total flavonoids were measured by a colorimetric assay modified by Boukhris, Simmonds, Sayadi, & Bouaziz [12]. An aliquot (1 mL) of the diluted sample or standard solution of quercetin (20, 40, 60, 80, and 100 mg/L) was added to a 10 mL volumetric flask containing 4 mL of H_2_O. At starting time, 0.3 mL of NaNO_2_ (5%, w/w) was added to the flask. After 5 min, 0.3 mL of AlCl_3_ (10% w/w) was added, successively at 6 min and 2 mL of NaOH (1M). Immediately, the reaction flask was diluted to volume with the addition of 2.4 mL of H_2_O and thoroughly mixed. The absorbance of the mixture, characterized by a pink color, was determined at 510 nm compared to a water control. Total flavonoids content (TFC) was expressed as mg quercetin equivalents (QE)/100 g of extract, using a calibration curve of quercetin.

### 2.7. Chromatographic Separation

The main compounds of flower OG extract were purified by chromatographic column with SiO_2_ as stationary phase and ethyl acetate/acetic acid/formic acid/water (10:0.5:0.5:1) as mobile phase. Precoated silica gel plates (silica gel 60 F_254_; thickness 0.25 mm; Merck) with the same above mobile phase were used to control the fraction separations: after development, the chromatogram was visualized at 254 nm and then was sprayed with natural products-polyethylene glycol (NP/PEG) reagent and visualized at 366 nm.

### 2.8. HPLC-DAD-MS Analysis

The analyses were performed using a JASCO modular HPLC system (Tokyo, Japan, model PU 2089) coupled to a diode array apparatus (MD 2010 Plus) and a FinniganMAT LCQ (ThermoQuest Corp./FinniganMAT; San Jose, CA) mass spectrometer module linked to an injection valve with a 20 *μ*l sampler loop. The column used was an Eclipse-PLUS-C18 (25 × 0.46 cm, i.d., 5 *μ*m) at a flow rate of 1.0 ml/min. The mobile phase consisted of solvent solution A (water/formic acid 99.5/0.5) and B (methanol/acetonitrile/formic acid 50/50/0.5). The gradient system adopted was chosen according to the molecules to analyze and characterized by six steps: (1) starting point at 90:10 v/v (A/B); (2) gradual changing to 70:30 v/v in 30 min; (3) progressive raise to 50:50 v/v up to 35 min; (4) increase to 30:70 v/v up to 40 min; (5) achievement of ratio 0:100 v/v up to 45 min; and (6) back to starting point (90:10 v/v) in 10 min. Injection volume was 40 *μ*L. The chromatograms were observed at 355 nm [13]. The mass experiments were carried out on a FinniganMAT LCQ (ThermoQuest Corp./FinniganMAT; San Jose, CA) mass spectrometer module, equipped with an ion trap mass analyzer and an ESI ion source electrospray, in negative ion mode. For ESI-MS and MS^2^ experiments, the parameters were set as follows: the capillary voltage was 3.5 kV, the nebulizer (N_2_) pressure was 20 psi, the capillary temperature was 300°C, the auxiliary gas (N_2_) flow was 9 l/min, and the skimmer voltage was 40 V. The mass spectrometer was operated in the negative ion mode in the m/z range 100–1500. Standard commercial molecules of quercetin 3-O-*β*-rutinoside (rutin), quercetin 3-O-*β*-glucuronide (miquelianin), isorhamnetin 3-O-*β*-rutinoside (narcissin), and isolated myricetin 3-O-*β*-glucuronide were used to confirm the experimental data.

The three commercial pure standards were also used to quantify these molecules in leaf and flower extracts through the construction of calibration curves with solutions of concentrations from 0.01 mg/mL to 0.4 mg/mL. The dried leaf and flower extracts were solubilized in hydroalcoholic solution (ethanol 70%) to obtain a final concentration of 4 mg/mL.

### 2.9. Spectrophotometric DPPH Assay

1 mg of OG extracts, quercetin 3-glucuronide, and trolox were dissolved into 0.900 mL of ethanol; then serial dilutions were assessed in order to obtain different concentrations. An aliquot (2.9 mL) of the ethanol solution of DPPH (4 mg/100 mL) was added to sample solution [14]. After 30 min incubation, in an orbital shaker at 200 rpm, in the dark at room temperature, the mixture was placed in a UV-Vis spectrophotometer (Helios Gamma Spectrophotometer) and the absorbance was read in triplicate against a blank at 517 nm. The DPPH inhibition in percent was determined by the following formula: IDPPH% = [1 - (A1/A2)] × 100, where A1 was the DPPH absorbance with the sample and A2 without the extract [14]. Trolox was used as positive control. The antioxidant activity of samples was expressed as IC_50_ (concentration providing DPPH 50% inhibition), calculated from inhibition curves obtained by plotting inhibition percentage against extracts concentration. All experiments were assessed in triplicate and values were reported as mean ± SD (Standard Deviation).

### 2.10. Oxidative Burst Test

Heparinized fresh venous blood sample was drawn from healthy volunteers and neutrophils were isolated [15]. Whole blood was added to Ficoll Paque; it was centrifuged for 30 minutes at 1500 rpm. After discarding the supernatant, red blood cells traces were lysed by mixing with hypotonic ammonium chloride solution (0.83% w/v). It was centrifuged again, and the neutrophils were washed with MHS (modified Hank's solution, pH 7.4) and resuspended at concentration of 10^7^ cells/mL in a MHS appropriate volume [16]. Anti-inflammatory activity was determined by using a modified* in vitro* assay based on the reduction of WST-1 in the presence of activated neutrophils [17]. Oxidative burst assay was determined in a total volume of 250 *μ*L MHS (pH 7.4) containing 10^7^ neutrophils/mL, 500 *μ*M WST-1, and various concentrations of test extracts. Control contained buffer, neutrophils, and WST-1. All compounds were equilibrated at 37°C and the reaction was initiated by adding opsonized Zymosan A (15 mg/mL), which was prepared by mixing it with human pooled serum, followed by centrifugation at 3000 rpm and pellet was suspended in phosphate buffer solution (PBS). Absorbance was measured at 450 nm [16]. Aspirin was used as positive control since it is utilized as nonsteroidal anti-inflammatory drugs (NSAIDs) for treatment of several inflammatory diseases [18, 19]. DMSO was used as blank and the anti-inflammatory activity was expressed as produced superoxide anions inhibition percent.

### 2.11. Cell Viability Assay (Cytotoxicity)

Metabolically active cells reduce tetrazolium salts into colored formazan compounds. Therefore, tetrazolium salt-based colorimetric assays detect only viable cells. These sensitive assays can readily be performed in a microtiter plate with relatively few cells by using modified method [20]. In this study, the human isolated neutrophils (10^7^ cells/mL) were incubated with test extracts for 30 minutes and then WST-1 (250 *μ*M) was added and incubated in shaking water bath at 37°C for 3 h. The absorbance was measured at 450 nm. Triton X-100 (0.1% v/v) and DMSO (0.5% v/v) were used as controls showing 0.00% and 100% cell viability, respectively [16]. The OD (optical density) is the mean of five replicates. Cell viability percent was calculated by using the following formula:

(1) $$\begin{array}{c} \text{\% Cell viability}=\left(\;\frac{{OD}_{TEST}}{{OD}_{DMSO}}\;\right)\times 100 \end{array}$$

### 2.12. Data Analysis

The results are expressed as mean ± SD. Student's t-test and one-way analysis of variance (ANOVA), where applicable, were used to analyze level of statistical significance between groups. P value less than 0.05 was considered statistically significant.

## 3. Results and Discussion

### 3.1. Chemical Characterization

**Figure 1:**
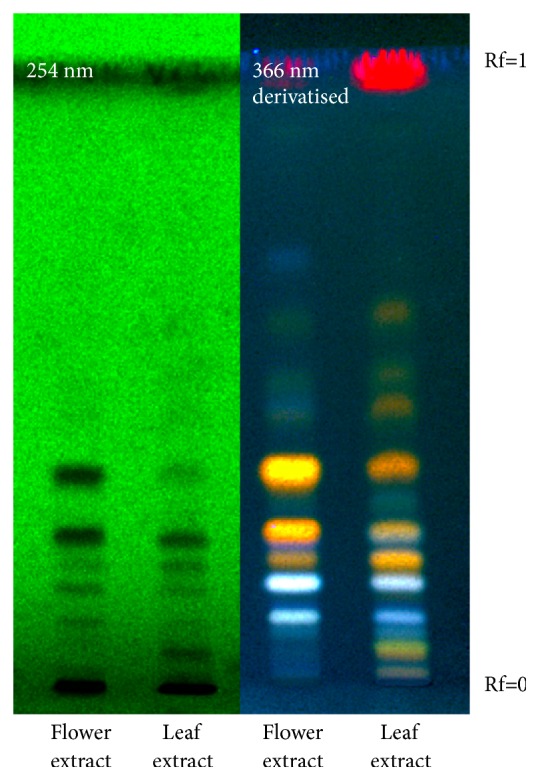
HPTLC of two extracts, visualized at 254 nm before derivatization and at 366 nm after derivatization. F. extract: flower extract and L. extract: leaf extract.

With the aim of chemically characterizing leaves and flowers of OG for determining their bioactive constituents, a qualitative preliminary screening was performed on the two OG hydroalcoholic extracts. The choice of the hydroalcoholic solvent has been guided by the ethnobotanical knowledge about OG traditional preparations [6] and by the need to perform an effective strategy to extract phenols, polyphenols, and flavonoids, reported as abundant secondary metabolites in the studied plant species belonging to Proteaceae family. In the first preliminary approach with HPTLC analyses, OG flower extract (F. extract) showed two main spots (Rf = 0.3 and 0.4) at 254 nm, evidenced also as orange fractions at 366 nm after selective derivatization for flavonoids class. The leaf extract (L. extract) showed instead a more complex composition and a red spot at 366 nm on the front of the solvent due to chlorophylls (Figure 1).

**Figure 2:**
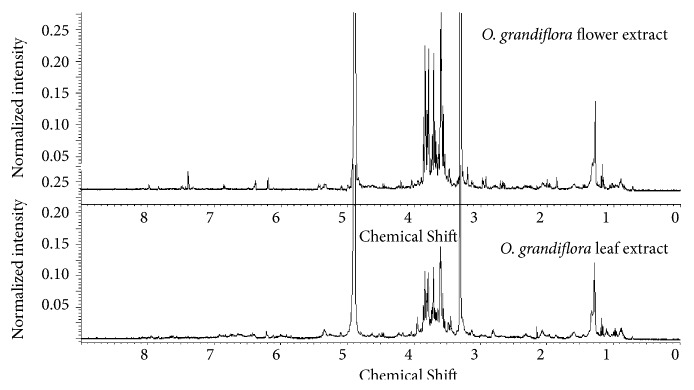
^1^HNMR of two crude extracts. The zone of 6-8 ppm is typical for signals of phenolic protons (flavonoids), while the zone between 3 and 4 ppm evidences for both extracts a relevant abundance of sugars.

The proton nuclear magnetic resonance is an appropriate technique to make a chemical fingerprinting, allowing at the same time a semiquantitative evaluation of a complex mixture [21]. In fact, the high signals between 3 and 4 ppm evidenced for both extracts the relevant abundance of sugars, probably corresponding to observable spots at Rf = 0 in HPTLC test at 254 nm, with respect to the flavonoids that showed resonances of lower intensity in the range of 6-8 ppm. Moreover, the flower mixture highlighted a simpler profile than to the leaf one at 6-8 ppm, confirming the results visualized with HPTLC assay (Figure 2).

Quantitative flavonoid content, measured by a colorimetric assay modified by Boukhris et al. [12], suggested that OG possessed appreciable quantity. Leaf extract was found to contain higher amounts of flavonoids than flower extract: 406.1 ± 1.73 mg of QE/g for the first and 158.5 ± 1.53 mg of QE/g for the second. A direct relationship was observed comparing these data with the extraction yields (%): 3.02% and 1.82% (% w/w), respectively, for leaf and flower extracts.

A separation through silica gel chromatographic column allowed successfully separating the two main fractions of* O. grandiflora* flower extract that successively were characterized by ^1^H, ^13^C, and bidimensional NMR experiments to determine their chemical structure. COSY and HMBC experiments confirmed the aglycone structure of quercetin, evidencing the coupling of protons 2', 5', 6', and 5, 7 at short and long distance, respectively; likewise, for myricetin structure the coupling of protons 2', 6' and 5, 7 was observed. As previously indicated by Rashed, Said, Abdo, and Selim [22] and Moon, Tsushida, Nakahara, and Terao [23] our mono- and bidimensional spectra showed also the couplings on the sugar moiety linked to quercetin and myricetin, respectively, allowing the complete elucidation of the chemical structures: quercetin 3-O-*β*-glucuronide and myricetin 3-O-*β*-glucuronide. Based on the results deriving by bidimensional spectra, we reported the assignments of chemical shifts to relative protons and carbons in ^1^H, ^13^C NMR experiments.

#### 3.1.1. Quercetin 3-O-*β*-Glucuronide

^1^H-NMR (CD_3_OD, 400 MHz): *δ* ppm 7.67 (1H, d, J = 2.1 Hz, H-2'), 7.62 (1H, dd, J = 8,4, 2.1 Hz, H-6'), 6.86 (1H, d, J = 8.4 Hz, H-5'), 6.40 (1H, d, J = 2.1 Hz, H-8), 6.20 (1H, d, J = 2.1 Hz, H-6), 5.31 (1H, d, J = 7.5 Hz, H-1”), 3.57 (1H, m, H-4”), 3.55 (1H, m, H-5”), 3.51 (1H, d, J = 7.5 Hz, 2”), 3.48 (1H, m, H-3”). ^13^C-NMR (CD_3_OD, 400 MHz): *δ* 178.05 (C-4), 175.00 (C- 6”), 164.64 (C-7), 161.60 (C-5), 158.80 (C-2), 158.08 (C-9), 148.43 (C-4'), 144.49 (C-3'), 134.96 (C-3), 121.37 (C-6', C1'), 116.56 (C-2'), 114.73 (C-5'), 102.94 (C- 10), 101.09 (C-1”), 98.67 (C-6), 93.26 (C-8), 76.61 (C-3”), 76.23 (C-5”), 74.10 (C-2”), 71.93 (C-4”).

#### 3.1.2. Myricetin 3-O-*β*-Glucuronide

^1^H-NMR (CD_3_OD, 400 MHz): *δ* ppm 7.37 (2H, s, H-2', 6'), 6.40 (1H, d, J = 2.1 Hz, H-8), 6.20 (1H, d, J = 2.1 Hz, H-6), 5.28 (1H, d, J = 7.5 Hz, H-1”), 3.57 (1H, m, H-4”), 3.55 (1H, m, H-5”), 3.51 (1H, d, J = 7.5 Hz, 2”), 3.48 (1H, m, H-3”). ^13^C-NMR (CD_3_OD, 400 MHz):*δ* 177.98 (C-4), 175.00 (C- 6”), 164.37 (C-7), 161.56 (C-5), 158.35 (C-2), 157.87 (C-9), 145.06 (C-3',5'), 136.59 (C-4'), 134.61 (C-3), 120.13 (C-1'), 108.56 (C-2',6'), 104.21 (C- 10), 103.18 (C-1”), 98.51 (C-6), 93.25 (C-8), 76.68 (C-3”), 76.40 (C-5”), 74.06 (C-2”), 71.92 (C-4”).

**Figure 3:**
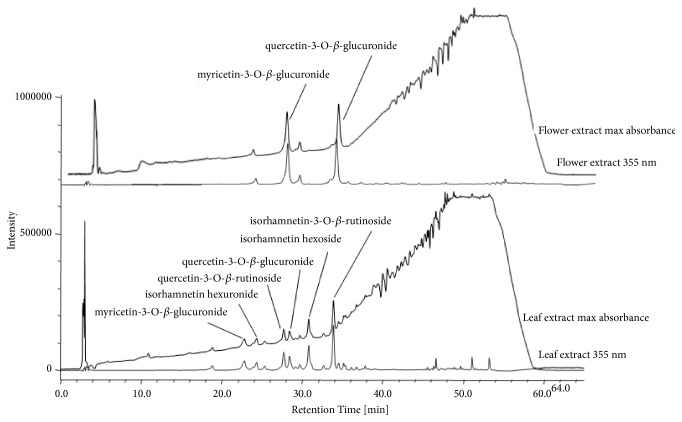
RP-HPLC-DAD chromatograms of leaf and flower extracts. For each extract the chromatogram recorded at the maximum absorbance (top) and at 355 nm (below) was reported.

The two extracts were then analyzed by HPLC-DAD and HPLC-MS. From the data analysis it results that quercetin 3-O-*β*-glucuronide and myricetin 3-O-*β*-glucuronide were the main components of flower extract, while isorhamnetin 3-O-rutinoside and quercetin 3-O-rutinoside resulted in the most abundant compounds of leaf extract. The results of the identification and the quantification of flavonoids are shown in Table 1, while the relative chromatograms are reported in Figure 3.

The ion negative mode ESI-MS gave information about the molar mass, MS^2^ spectra indicated the cleavage of the glycosidic bond that led to the elimination of the sugar residue, resulting in strong fragments at m/z 317, 315, and 301, corresponding to myricetin, isorhamnetin, and quercetin, respectively. The MS and UV spectra were in accord with literature data and pure compounds were used to confirm the identification [24, 25].

### 3.2. DPPH Radical Scavenging Activity

The oxidative stress plays a crucial role in the development and perpetuation of inflammation [26]. In order of demonstrating the traditional use of OG as anti-inflammatory crude drug we performed a preliminary evaluation of antioxidant activity through DPPH assay to support the data of more specific anti-inflammatory tests. As shown in Table 2, the leaf extract highlighted an interesting scavenging ability if compared to trolox, used as positive control. Quercetin 3-glucuronide, used as potential bioactive compound, was the best performing compound with IC_50_ values even slightly better than that of trolox.

Regarding the antioxidant activity, it must be emphasized that quercetin and quercetin glycosides are well known as radical scavenging molecules: chlorophylls also, evidenced in leaf extract through HPTLC analysis and contribute to the efficacy [27, 28]. Therefore, our results confirmed the literature data and could suggest potential application of OG as antioxidant food supplement or agent to control oxidative stress linked to several diseases.

### 3.3. Evaluation of Cytotoxicity and In Vitro Anti-Inflammatory Activity

The inhibitory effect of different concentrations of OG leaf and flower extracts on isolated activated neutrophils model using stable tetrazolium salt (WST-1) is summarized in Table 3.

Both extracts (from 12.5 to 200 *μ*g/mL) showed significant inhibition of inflammation on isolated neutrophils (activated with opsonized Zymosan A) in a dose-dependent manner. The* in vitro* anti-inflammatory activity was comparable to the aspirin, a reference anti-inflammatory drug at the same dose levels of extracts. A significant difference in the inflammatory inhibition was observed in case of leaf extract when compared with the flower extract at every tested concentration.

The cytotoxicity was evaluated in terms of cell viability using freshly isolated neutrophils as cellular model. The results of the cytotoxicity are shown in Table 4.

The cell viability decreased with the increase of the concentration of OG leaf and flower extracts. The cytotoxicity of OG leaf extract on isolated neutrophils is remarkable.

The results of the inflammatory inhibition assay, with particular reference to the oxidative burst pathway, showed a proportional relation between the concentration of OG crude drugs extracts and the anti-inflammatory activity. Genus* Oreocallis* has not been widely studied for its biological activity and phytochemicals. In this context, the present research, highlighting the noteworthy inhibition of the oxidative burst pathway, and the high amount of flavonoids in the OG extracts (especially quercetin derivatives) consolidated the idea that this particular class of compounds could be responsible for the anti-inflammatory activity of the studied phytocomplexes. Flavonoids have various biological activities, which are mainly related to its ability to inhibit enzymes and its effects on immune responses [29]. The pharmacological effects of quercetin, such as its antioxidant, anti-inflammatory, antiallergic, antiaging, and anticancer activities, as well as its regulating effect on interleukin- (IL-) 6, IL-8, tumor necrosis factor (TNF), histamine, and tryptase release in mast cells, are well known [30].

Recently, Lesjak et al. [31] demonstrated that quercetin and its derivatives show a notable concentration-dependent inhibitory potential towards synthesis of inflammatory mediators, which was comparable with aspirin, drug used as reference in that study. Likewise, Lesjak et al. [31] established that overall order of anti-inflammatory activity to quercetin derivatives is quercetin 4′-methyl ether > quercetin = quercetin-3,4′-di-O-glucoside > isorhamnetin = quercetin-3-O-glucuronide > isorhamnetin-3-O-glucoside > quercetin-3,5,7,3′,4′-penthamethylether, and it set up clear that the number of free hydroxyl groups was not the only characteristic which could define potential of quercetin conjugates to inhibit COX-1 and 12-LOX pathway of AA metabolism [31]. In the same sense, other researches mention that the number of free hydroxyl groups of flavonoids is not directly proportional with their anti-inflammatory activity, specifically for their COX-2 inhibition potential [32–34]. The exact mechanism of how flavonoids inhibit COX and LOX activity is not known [35].

When comparing the anti-inflammatory activity of OG flower extract with the anti-inflammatory capacity of aspirin, they do not have a statistically significant difference (t-Student p<0.072); on the other side, the potential of OG leaf extract as an anti-inflammatory agent is evident (around 97% inflammatory inhibition) and it differs to aspirin (t-Student p<0.002). Nevertheless, it should be stressed that the concentrations of OG leaf extract considered are highly cytotoxic and therefore it could be possible that the anti-inflammatory data might be altered by this bioactivity, even so, the traditional medicine reports the traditional use of leaf extract. It should be interesting to explore the anti-inflammatory potential of leaf extracts also at lower and noncytotoxic concentrations. In light of this consideration, the flower extract appears to be more interesting for health applications, in particular for those concentrations that did not evidence cytotoxicity (1.56-6.25 *μ*g/mL). In fact, the OG flower extract showed an anti-inflammatory activity equivalent to that of aspirin with a considerable advantage, since the cytotoxicity of the aspirin was much higher than that of flower extract (Table 4), which implies an important safety of use of this crude drug and makes it a relatively safe alternative for its use as an anti-inflammatory. Finally, the research carried out is a contribution to valorize the forgotten ancient use of this resource (for example, the preparation of “horchata” beverage), currently underutilized. The results of this study could become an opportunity for the local development of the communities through the planting, conservation and rational use of this species and, in this way, improve their economy.

## 4. Conclusions

In this work, for the first time, flavonoids have been identified in OG leaf and flower hydroalcoholic extracts. In particular, quercetin 3-O-*β*-glucuronide and myricetin 3-O-*β*-glucuronide have been isolated through silica gel chromatography and fully characterized through NMR and mass spectrometry. In the leaf extract other two flavonoids were identified and quantified by HPLC-MS-DAD: quercetin 3-O-*β*-rutinoside (rutin) and isorhamnetin 3-O-*β*-rutinoside (narcissin). Moreover, based on the* in vitro *results of this study, it can be concluded that OG is a very interesting source of natural antioxidant and anti-inflammatory compounds that could be used to prevent many chronic disorders and could be suggested as food supplement. Further in depth investigations on flavonoids responsible for the biological properties should be performed as well as experiments to discover their potential mechanism of action as single molecules and their mixtures.

## Figures and Tables

**Table 1:** Flavonoids identified in leaf extract of *O. grandiflora* by HPLC-UV/DAD, HPLC-ESI-MS, and MS^2^.

Peak number	Compound^a^	Flower extract^d^ mg/g d.e. (mg/g drug) ^e^	Leaf extract mg/g d.e. (mg/g drug)	UV_max_ (nm)	[M-H]^−^ (m/z)	MS^2^ (m/z) base peak
1	Myricetin 3-O-*β*-glucuronide^b^	d	d	255,355	493	317
2	Isorhamnetin hexuronide	nd	d	251,351	491	315, 299
3	Quercetin 3-O-rutinoside^c^	nd	15.6 ± 0.4 (0.47 ± 0.01)	255,355	609	301
4	Quercetin 3-O-*β*-glucuronide^c^	34.7±0.6 (0.63 ± 0.01)	5.8 ± 0.1 (0.18 ± 0.01)	255,355	477	301
5	Isorhamnetin hexoside	nd	d	255,355	477	315, 299
6	Isorhamnetin 3-O-rutinoside^c^	nd	21.1 ± 1.2 (0.64 ± 0.01)	255,355	623	315, 299

^a^Compounds are listed in order of elution, ^b^confirmed with injection of isolated compound, ^c^confirmed with commercially standard compound, ^d^detected = d, not detected = nd, and ^e^d.e. = dried extract.

**Table 2:** DPPH activity of the two extracts and quercetin 3-glucuronide.

**Samples**	**IC**_**50**_ ** **μ**g/mL**
*O. grandiflora *flower extract	14.39 ± 1.43
*O. grandiflora *leaf extract	6.69 ± 1.39
quercetin 3-glucuronide	3.73 ± 0.11
trolox	4.02 ± 0.25

**Table 3:** *In vitro* anti-inflammatory effect of OG leaf and flower extracts by isolated neutrophils model using stable tetrazolium salt (WST-1).

Concentration (*μ*g/mL)	Inflammatory Inhibition (%)		
	Leaf extract	Flower extract	Aspirin
1.56	39.87 ± 1.64^*∗*^	43.42 ± 1.21	36.21 ± 1.43
3.12	48.32 ± 1.18^*∗*^	46.67 ± 1.39	39.14 ± 1.15
6.25	56.76 ± 0.83^*∗*^	51.31 ± 1.77	42.25 ± 0.86
12.5	62.17 ± 0.45^*∗*^	54.04 ± 1.40	57.43 ± 0.21
25	74.41 ± 0.71^*∗*^	57.75 ± 0.47	61.54 ± 0.33
50	87.83 ± 1.11^*∗*^	60.19 ± 0.64	65.31 ± 0.18
100	88.80 ± 0.61^*∗*^	61.38 ± 0.80	69.85 ± 0.25
200	97.18 ± 1.85^*∗*^	68.27 ± 2.88	74.11 ± 0.11
IC_50_	4.08 ± 0.07	5.87 ± 1.48	8.85 ± 0.79

Values are mean ± SD, n=5. Significant values, P<0.05, using Student's t-test; leaf extract versus flower extract.

**Table 4:** Cell viability percentage of OG leaf and flower extracts at different concentrations.

Concentration (*μ*g/mL)	Cell viability (%)		
	Leaf extract	Flower extract	Aspirin
1.56	83.85 ± 1.17	99.97 ± 0.98^*∗*^	68.41 ± 0.74
3.12	62.49 ± 0.85	97.22 ± 2.33^*∗*^	61.68 ± 0.86
6.25	46.15 ± 0.77	93.31 ± 2.12^*∗*^	54.53 ± 0.67
12.5	35.58 ± 0.53	87.80 ± 2.26^*∗*^	47.70 ± 0.54
25	32.50 ± 0.81	61.54 ± 2.03^*∗*^	43.64 ± 0.49
50	14.23 ± 0.86	53.85 ± 1.89^*∗*^	39.59 ± 0.25
100	11.92 ± 0.44	48.28 ± 1.79^*∗*^	34.41 ± 0.37
200	11.73 ± 0.57	40.58 ± 0.84^*∗*^	31.48 ± 0.22

Values are mean ± SD, n=5. ^*∗*^Significant values, P<0.05, using Student's t-test; leaf extract versus flower extract. *Note.* Triton X-100 (0.1%) was used as negative control, showing 0.00% cell viability.

## Data Availability

The data of chemical characterization and bioactivity evaluation used to support the findings of this study are included within the article.
